# Novel molecular approach to define pest species status and tritrophic interactions from historical *Bemisia* specimens

**DOI:** 10.1038/s41598-017-00528-7

**Published:** 2017-03-27

**Authors:** W. T. Tay, S. Elfekih, A. Polaszek, L. N. Court, G. A. Evans, K. H. J. Gordon, P. J. De Barro

**Affiliations:** 1 CSIRO, Black Mountain Laboratories, Clunies Ross Street, Canberra, ACT 2601 Australia; 2 Natural History Museum, London, UK; 3 USDA APHIS NIS, BARC-West, Beltsville, Maryland United States of America; 4 CSIRO, Brisbane, 4001 Queensland Australia

## Abstract

Museum specimens represent valuable genomic resources for understanding host-endosymbiont/parasitoid evolutionary relationships, resolving species complexes and nomenclatural problems. However, museum collections suffer DNA degradation, making them challenging for molecular-based studies. Here, the mitogenomes of a single 1912 Sri Lankan *Bemisia emiliae* cotype puparium, and of a 1942 Japanese *Bemisia* puparium are characterised using a Next-Generation Sequencing approach. Whiteflies are small sap-sucking insects including *B*. *tabaci* pest species complex. *Bemisia emiliae*’s draft mitogenome showed a high degree of homology with published *B*. *tabaci* mitogenomes, and exhibited 98–100% partial mitochondrial DNA Cytochrome Oxidase I (mtCOI) gene identity with the *B*. *tabaci* species known as Asia II-7. The partial mtCOI gene of the Japanese specimen shared 99% sequence identity with the *Bemisia* ‘JpL’ genetic group. Metagenomic analysis identified bacterial sequences in both *Bemisia* specimens, while hymenopteran sequences were also identified in the Japanese *Bemisia* puparium, including complete mtCOI and rRNA genes, and various partial mtDNA genes. At 88–90% mtCOI sequence identity to Aphelinidae wasps, we concluded that the 1942 *Bemisia* nymph was parasitized by an *Eretmocerus* parasitoid wasp. Our approach enables the characterisation of genomes and associated metagenomic communities of museum specimens using 1.5 ng gDNA, and to infer historical tritrophic relationships in *Bemisia* whiteflies.

## Introduction

Since its description by Gennadius in 1889, the taxonomy of the whitefly *Bemisia tabaci* has proven a challenge. There had been various significant taxonomic revisions^1–3^ within this nominal species, but the lack of unique morphological features associated with these led to all being synonymised under the name ‘*B*. *tabaci*’. The confusing nomenclature was revised using allozyme and DNA markers that indicated substantial sub-clustering, eventually giving rise to the biotype concept. This has more recently been superseded, by thorough integration of sequence and biological data which demonstrated that *B*. *tabaci* is in fact a complex of more than 43 cryptic biological species^4^.

The recognition that *B*. *tabaci* is a species complex presented a further challenge, which is to link collection specimens (all tagged with the nomenclature at the time of identification) with the newly adopted genetically-based structure^5^. Tay *et al.* ^6^ determined the identity of the original 1889 Gennadius *B*. *tabaci* whitefly specimen through Sanger sequencing of multiple PCR products. Characterisation of the partial mitochondrial DNA cytochrome oxidase subunit I (mtCOI) gene from a single museum individual originally collected by Gennadius in 1889 subsequently showed that the Mediterranean (‘MED’) member of the complex was the true ‘*B*. *tabaci*’. Since then, there have been no further studies using museum specimens to address taxonomic issues in the remaining members of the *B*. *tabaci* complex.

One of the most important concerns in working with historical specimens is the finite amount of material available. PCR primers for the partial mtCOI region^7^ can be inefficient, due to the large diversity within *B*. *tabaci* clades^4, 8^, and multiple PCR amplification attempts can rapidly deplete the sample with no guarantee of success. Attempts to overcome this have involved replacing the commonly-used universal primers developed by Simon *et al.* ^9^ with ones designed for a specific clade. For instance, partial mtCOI gene diversity ‘within species’ typically ranges between 0 to 3.4%, while it ranges from 3.1 to 5.5% between species within clades (e.g., MEAM1 vs MED), to 15.7% to 16.5% for species between clades (i.e., *B*. *tabaci* SSA4 vs MEAM1)^5^. However, in addition to primer design, factors such as the poor quality and low yield of the genomic DNA (gDNA) due to the specimen size, preservation methods and the age of the material, have all contributed to the difficulty of mtCOI genotyping. Work on historical *Bemisia* samples would therefore greatly benefit from novel, more efficient technologies.

Next-generation sequencing (NGS) approaches are now routinely used to characterise mitogenomes from individual specimens (e.g., Arnemann *et al.* ^10^), and including from a single *Bemisia* individual^11^, although the method of Tay *et al.* ^11^ used at least 18 ng of double stranded gDNA for NGS library construction. Recently, Timmermans *et al.* ^12^ demonstrated that a large volume of both nuclear and mitochondrial DNA data could be generated and captured from single historical insect individuals using NGS platform. These NGS approaches not only bypassed primer binding efficacy issues, but offer vastly more effective utilisation of gDNA from individual specimens of interest. The NGS platform therefore represents an attractive option for studying historical *Bemisia* specimens, and will enable us to relate these individuals to our emerging understanding of challenging species complexes, associated metagenomics compositions, and host-parasitoid interactions.

Here, we describe the draft mitogenomes of two historical *Bemisia* specimens, that of a 104 year-old *B*. *emiliae* Corbett 1926, previously synonymised with *B*. *tabaci* ^2^, and a 74 year-old whitefly specimen identified as ‘*B*. *tabaci*’ from Japan in 1942, prepared using a Nextera XT DNA library and sequenced using an Illumina MiSeq sequencer. The high-throughput sequence data allowed us to conduct a comparative genomics analysis between *Bemisia* species, to explore their metagenomic communities, and provide insights into the diversity of *Bemisia* parasitoid species.

## Results

**Figure 1:**
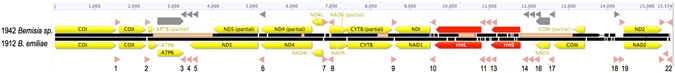
Diagrammatic representation of the complete mitochondrial DNA genome of *Bemisia emiliae* (black bar), showing orientation of protein coding genes (PCGs), tRNAs and rRNAs, with the mitochondrial DNA cytochrome oxidase (mtCOI) gene arbitrarily selected as a starting point. The mitochondrial DNA genome of the 1942 Japanese *Bemisia* sp. is aligned against that of *B*. *emiliae*, and gaps are introduced for alignment purposes. Predicted tRNAs, and partial PCGs of the 1942 *Bemisia* sp. are as detailed in Supplementary Table 1. Missing tRNAs and PCGs are shown in grey with corresponding orange segments on the mtDNA genome schematic diagram of the 1942 *Bemisia* species.

The single 1912 *B*. *emiliae* 4^th^ instar nymph (“puparium”) yielded a total of 4.62ng of double stranded gDNA, and the 1942 ‘*B*. *tabaci*’ 4^th^ instar from Japan yielded 27.85 ng of double stranded gDNA. The Illumina MiSeq run generated 4,520,563 pair-end sequence reads (i.e., 9,041,126 reads) for *B*. *emiliae*, and 2,906,587 pair-end sequence reads (i.e., 5,813,174 reads) for the Japanese ‘*B*. *tabaci*’. Pair-end sequences from both the 1912 *B*. *emiliae* and the Japanese ‘*B*. *tabaci*’ supported the mitogenomes as circular molecules. The draft mitogenomes of both museum specimens were assembled using the mitogenome of *B*. *tabaci* Asia I (KJ778614) as a reference. We recovered a complete mitogenome for *B*. *emiliae* (15,515 bp from 37,089 reads) (GenBank KX714967) and a partial mitogenome (GenBank KX714968) for the 1942 ‘*B*. *tabaci*’ individual from 2,607 NGS genomic fragments (Fig. 1). An estimated 3,635 bps distributed across six regions of the mitogenome were missing from the 1942 *Bemisia* specimen, impacting eight protein coding genes (PCGs), eight tRNAs, and one rRNA (Fig. 1; Supplementary Table 1). Alignment with the assembled *B*. *tabaci* Asia II-7 (Fig. 1, Supplementary Table 1) indicated that these missing mitogenome regions included regions spanning partial ATP8 to partial ND5, part of Cyt *b*, part of the large subunit rRNA and small subunit rRNA, missing tRNA^Asn^, tRNA^Arg^, tRNA^Ala^, ND3, tRNA^Gly^, and also part of COIII. Gene orientation and gene order between the 1912 and 1942 *Bemisia* specimens were identical overall and as confirmed via the *De Novo* Assemble algorithm within Geneious version 8.0.5 (Biomatters Ltd, Auckland, NZ) (data not shown), although gene orientation for ATP6, ND3, and the missing tRNAs for the Japanese *Bemisia* specimen could not be ascertained. The assembled partial mitogenome of the Japanese *Bemisia* consisted of 2,255 sequences, with an estimated sequence genome length of 15,214 bp.

The 1912 *B*. *emiliae* cotype specimen had a 100% partial mtCOI (657 bp) sequence identity to three members (i.e., GQ139492, AJ748378, DQ174523) of the *B*. *tabaci* Asia II-7 clade, and ranged between 98% (AY686075) and 99% sequence identity (AM408899, DQ174523, AJ748372, DQ116650, DQ116661, DQ116660, DQ174521, AJ748375, DQ116662, AY686064) with other *B*. *tabaci* Asia II-7 members. All reported Asia II-7 members were of Asian origin (e.g., India, Taiwan, China). Similarly, based on partial mtCOI sequence (777 bp) identity, the 1942 *Bemisia* specimen matched 99% with members of the *Bemisia* genetic group of ‘JpL’^13^ (GenBank accession numbers AB308111, AB308114-AB308119, AB240967, accessed 02-Jun-2016), all of which are from Japan. Phylogenetic analysis^5, 13^ based on the same partial mtCOI gene region indicated a basal position of the ‘JpL’ genetic group to the ‘*B*. *tabaci*’ species complex, providing support that this 1942 Japanese ‘*B*. *tabaci*’ was likely a non-‘*tabaci*’ species. Its basal position clusters with other members of *Bemisia* that were, until recently, members of *Lipaleyrodes*, which was synonymised with *Bemisia* in 2009^14^.

Metagenomics analysis showed that 90.4% of *B*. *emiliae* sequences was of bacterial origin, of which 84.3% belonged to the Gram-negative Proteobacteria phylum, and only 6% to Arthropoda. This contrasted significantly with the metagenomic compositions of the 1942 *Bemisia* individual where only 26% was of bacterial origin and 61% of arthropod origin (Table 1). Interestingly, both *B*. *emiliae* and the 1942 *Bemisia* specimens had low (0.2% and 0.1%, respectively) sequences that matched to *Bemisia*, and is reflective of the absence of a *Bemisia* genome. A total of 1.5% of sequences from *B*. *emiliae* matched sequences corresponding to Hymenoptera (i.e., *Nasonia*, *Harpegnathos*, *Camponotus*, *Apis*), while this was 36.5% for the 1942 *Bemisia* specimen (Table 1). We detected the primary (P)-endosymbiont *Candidatus Portiera aleyrodidarum* in both hosts, as well as similar proportions of the facultative secondary (S)-endobacteria *Cand*. *Hamiltonella* and *Rickettsia*, and a higher proportion of *Wolbachia* was detected in the 1942 *Bemisia* than in *B*. *emiliae*. Finally, both *Arsenophonus* (Enterobacteriaceae) and *Cand*. *Cardinium* (Bacteroidaceae) were detected only in *B*. *emiliae* (Table 1).

**Table 1:** MiSeq input and post quality control (QC) data for metagenomic analysis via MG-Rast standard pipeline for the 1912 *B*. *emiliae* specimen (MG-Rast accession number: 4681440.3) and the 1942 Japanese *Bemisia* species (MG-Rast accession number: 4690946.3).

	1912 *B*. *emiliae*	%	1942 *Bemisia* sp.	%
Up-load count (bp)	950,823,796		870,198,516	
up-load seq count	9,041,126		5,813,174	
Upload: Mean Sequence Length (bp)	105 ± 54		149 ± 56	
Post QC: Count (bp)	848,681,218	87.6	839,130,417	95.4
Post QC: Sequences Count	7,921,154		5,544,879	
Post QC: Mean Sequence Length (bp)	107 ± 54		151 ± 55	
**Taxonomic Hits Distribution**				
Bacteria	860,366	90.4*	75,320	26
Proteobacteria	803,377	84.3*	58,567	20.2
**Enterobacteriaceae**	75,341	7.9	13,319	4.6
*Cand*. *Hamiltonella*	1,613	0.2	491	0.2
*Cand*. *Portiera aleyrodidarum*	545	0.06	1,228	0.4
*Arsenophonus*	539	0.06	n/a	n/a
**Bacteroidaceae**	1,642	0.2	703	0.2
*Cand*. *Cardinium*	87	<0.01	n/a	n/a
**Alphaproteobacteria**	142,248	14.9*	11,640	4.0
*Rickettsia*	1,527	0.2	915	0.3
*Wolbachia*	1,427	0.2	3,743	1.3*
**Comamonadaceae**				
*Acidovorax*	291,123	30.1*	6,903	2.4
**Eukaryota**	86,636	9.1	211,856	73.1*
Arthropoda	57,242	6.1	176,903	61.0*
*Bemisia*	2,088	0.2	429	0.1
*Nasonia*	5,527	0.6	64,603	22.1*
*Harpegnathos*	3,064	0.3	15,369	5.2*
*Camponotus*	2,858	0.3	17,345	5.9*
*Apis*	2,931	0.3	9,546	3.3*
Viruses	2,036	0.2	1,522	0.5
Others	1,901	0.2	1,006	0.3

A summary of taxonomic hits for Bacteria, Eukaryota, Viruses are provided. Hit abundances that differed greatly between *B*. *emiliae* and the 1942 *Bemisia* sp. are indicated in by ‘*’.

**Figure 2:**
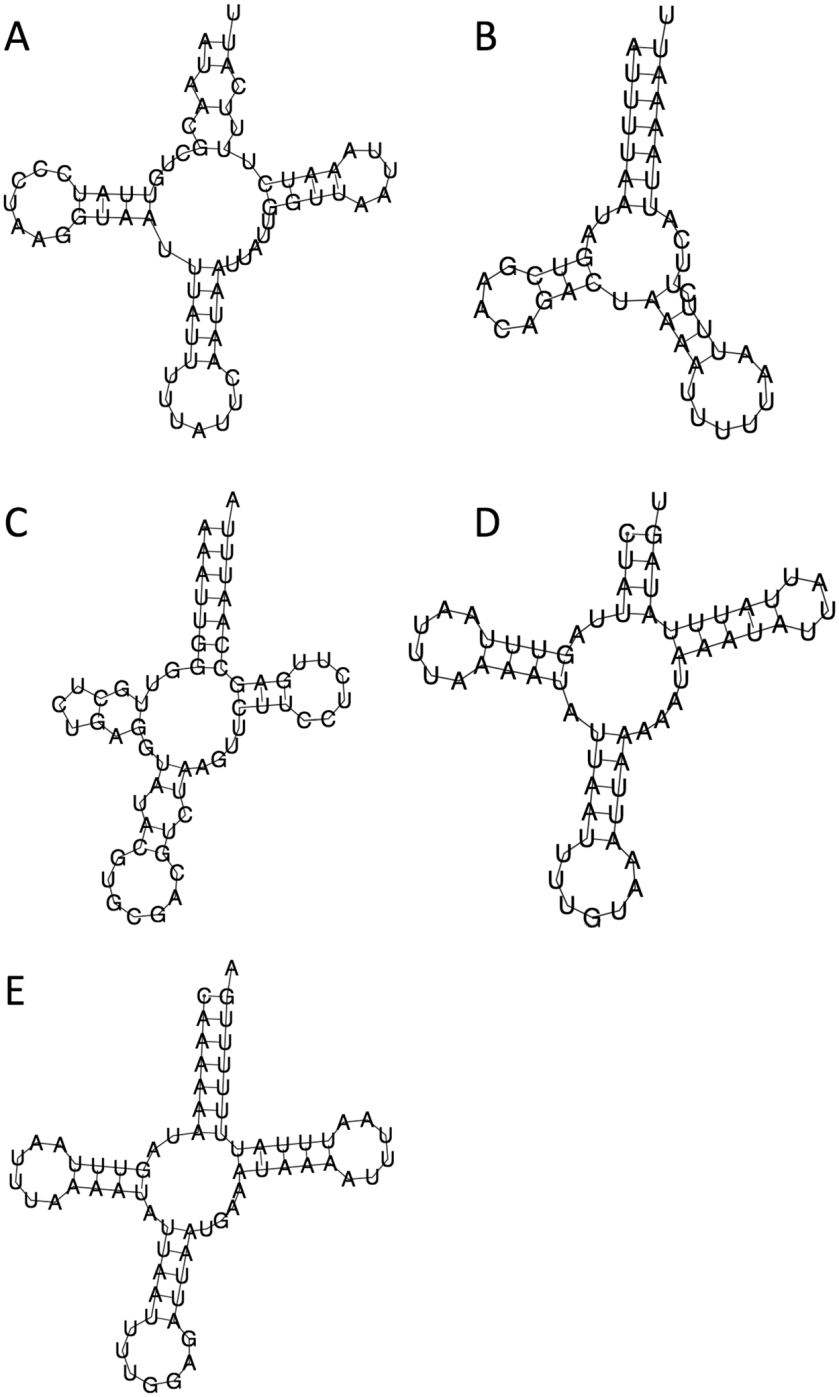
tRNA secondary structures of the hymenopteran parasitoid from the 1942 Japanese *Bemisia* sp. (**A**) The 68 bp tRNA^Met^ has an anticodon of (TAT) and is of the typical three clover leaf structure. (**B**) The tRNA^Lys^ is predicted to be 48 bp and has the unusual (TTT) anticodon and is lacking the TψC arm and loop. The tRNA^Arg^ is 60 bp in length and has the (GCG) anticodone (**C**); the tRNA^Thr^ (**D**) is 61 bp in length, and the tRNA^Pro^ is 69 bp in length (**E**), and have the (TGT) and (TGG) anticodons, respectively.

High proportions of sequences matching Hymenoptera (Table 2) in the 1942 *Bemisia* suggested either contamination, or alternatively parasitism by a hymenopteran parasitoid wasp. Mining the NGS sequence data successfully assembled a DNA contig of 8,951 bp (from 3,410 DNA fragments) that spanned the complete 16 S to 28 s ribosomal RNA (rRNA) genes and included the intergenic spacer (ITS) 1, the 5.8 s rRNA, and the ITS2 region (GenBank KX714966). A DNA contig of 1,926 bp (GenBank KX714952) was also assembled that included the complete mtDNA COI gene (1,536 bp; 512 amino acids), and two tRNAs (tRNA^Lys^ and tRNA^Met^) genes. The predicted tRNA^Met^ is 68 bp (Fig. 2A) and is within the typical tRNA lengths of 60–80 bp in Hymenoptera^15, 16^ and has the (TAT) anticodon. The tRNA^Lys^ has the (TTT) anticodon reported in various Hymenoptera species including species within the Chalcidoidea superfamily (e.g., *Encarsia formosa*), and may represent mutation from the ancestral state of (CTT) anticodon^17^. Interestingly, this tRNA^Lys^ is only predicted to be 48 bp in lengh and having a two-arms clover leaf secondary structure due to the absence of the TψC arm and loop (Fig. 2B). The prediction of this 48 bp tRNA^Lys^ secondary structure is unlikely to have been affected by contig assemblies using short NGS DNA fragments due to its centrally located position within multiple pair-end sequences, some of which are 200 bp long and extended across to the mtDNA COI gene (Supplementary Fig. 1). Though unusual, two-arms clover leaf tRNA secondary structures are known in diverse organisms from mammals (e.g., bovine^18^) to arthropods (e.g., trnS1 and trnS2 of *B*. *emiliae*, trnS2 of the 1942 Japanese *Bemisia* sp., this study; *Habronattus* jumping spiders^19^; Scelionidae parasitic Hymenoptera^20^), while the missing part of the TψC arm and loop is, to our knowledge, the first to be reported in an Aphelinidae parasitoid wasp species (see below). Various hymenopteran mtDNA partial genes were further assembled and included COII (424 bp, GenBank KX714953), ATP6 (289 bp, GenBank KX714954), ATP6-COIII (524 bp, KX714955), COIII (333 bp, GenGank KX714956; 200 bp, GenBank KX714957), NADH5 (232 bp, GenBank KX714958; 263 bp, GenBank KX714959; 411 bp, GenBank KX714960), NADH4 (155 bp, GenBank KX714961), NADH4-tRNA^Arg^ (Fig. 2C) (243 bp, 60 bp; GenBank KX714962), tRNA^Thr^-tRNA^Pro^-NADH6 (61 bp (Fig. 2D), 69 bp (Fig. 2E), 371 bp, GenBank KX714963), and NADH6-Cyt *b* (112 bp, 1,054 bp, GenBank KX714964) (Fig. 1).

**Table 2:** Annotations of the detected mitochondrial DNA (mtDNA) genes of a parasitoid *Eretmorcerus* sp. from the gDNA of the ‘1942’ Japanese *Bemisia* specimen.

	*Eretmocerus* sp. JAP1942	GenBank	nucleotide positions	Best matched organism	GenBank	Amino acid positions	Identity (%)	Number of reads
i	tRNA^Met^ (M)	KX714952	59..126					
ii	tRNA^Lys^ (K)	KX714952	186..233					
iii	COI	KX714952	362..1,879	*Nasonia longicornis*	ACH81769	11..516	82	168^A^
iv	COII	KX714953	3..422	*Megaphragma amalphitanum*	YP_009176325	67..206	74	40
v	ATP6	KX714954	102..314	*Philotrypesis pilosa*	AEG25310	1..67	32	306
vi	ATP6	KX714955	42..461	*Philotrypesis pilosa*	AEG25310	83..222	71	
vii	COIII	KX714955	469..522	*Philotrypesis pilosa*	AEG25309.1	2..19	78	139^B^
viii	COIII	KX714956	10..312	*Nasonia longicornis*	ACH81765	161..261	71	19
ix	COIII	KX714957	1..198	*Nasonia giraulti*	ACH81754	60..125	85	8
x	NADH5	KX714958	2..229	*Ceratosolen solmsi*	AEG67044.1	47..122	64	14
xi	NADH5	KX714959	2..262	*Nasonia giraulti*	ACH81759	193..279	83	10
xii	NADH5	KX714960	2..409	*Megaphragma amalphitanum*	YP_009176327	304..439	57	6
xiii	NADH4	KX714961	3..155	*Nasonia vitripennis*	ACH81738.1	268..318	65	4
xiv	NADH4	KX714962	34..243	*Nasonia vitripennis*	ACH81738.1	187..256	73	
xv	tRNA^Arg^ (R)	KX714962	278..337					198^C^
xvi	tRNA^Thr^ (T)	KX714963	1..61					
xvii	tRNA^Pro^ (P)	KX714963	67..135					
xviii	NADH6-0	KX714963	220.. 447	*Nasonia vitripennis*	ACH81751.1	29..104	49	8^D^
xiv	NADH6-0	KX714964	8..112	*Nasonia vitripennis*	ACH81751.1	148..182	51	
xx	Cyt *b*	KX714964	114..1,166	*Nasonia vitripennis*	ACH81741.1	2..347	76	74^E^

A total of 13 DNA fragments (GenBank accession numbers KX714952–KX714964) representing eight mtDNA protein coding genes and five tRNAs were detected. Amino acid positions of the best matched hymenopteran species within the Chalcidoidea superfamily and the percentage identity are also shown.

**Note**: (A) A total of 168 reads for the assembly of (i, ii, iii). (B) A total of 139 reads for the assembly of (vi, vii). (C) A total of 198 reads for the assembly of (xiv, xv). (D) A total of 8 reads for the assembly of (xv, xvi, xvii). (E) A total of 74 reads for the assembly of (xiv, xx).

Extensive local rearrangements and translocations within Hymenoptera mitogenomes are known^17^, and while gene orders for tRNA^Thr^-tRNA^Pro^-NADH6-Cyt *b* were similar to those reported for various parasitoid hymenopteran wasp species^20, 21^, identification of tRNA^Met^-tRNA^Lys^-COI, as well as the NADH4-tRNA^Arg^ gene orders would nevertheless suggest presence of novel mitogenome gene rearrangements in the parasitoid wasp, likely to be an *Eretmocerus* species (see below). Confirmation of such gene rearrangements will require future characterisation of the complete mtDNA genomes of *Eretmocerus* wasp species.

### Phylogenetic analysis of *Eretmocerus* species

**Figure 3:**
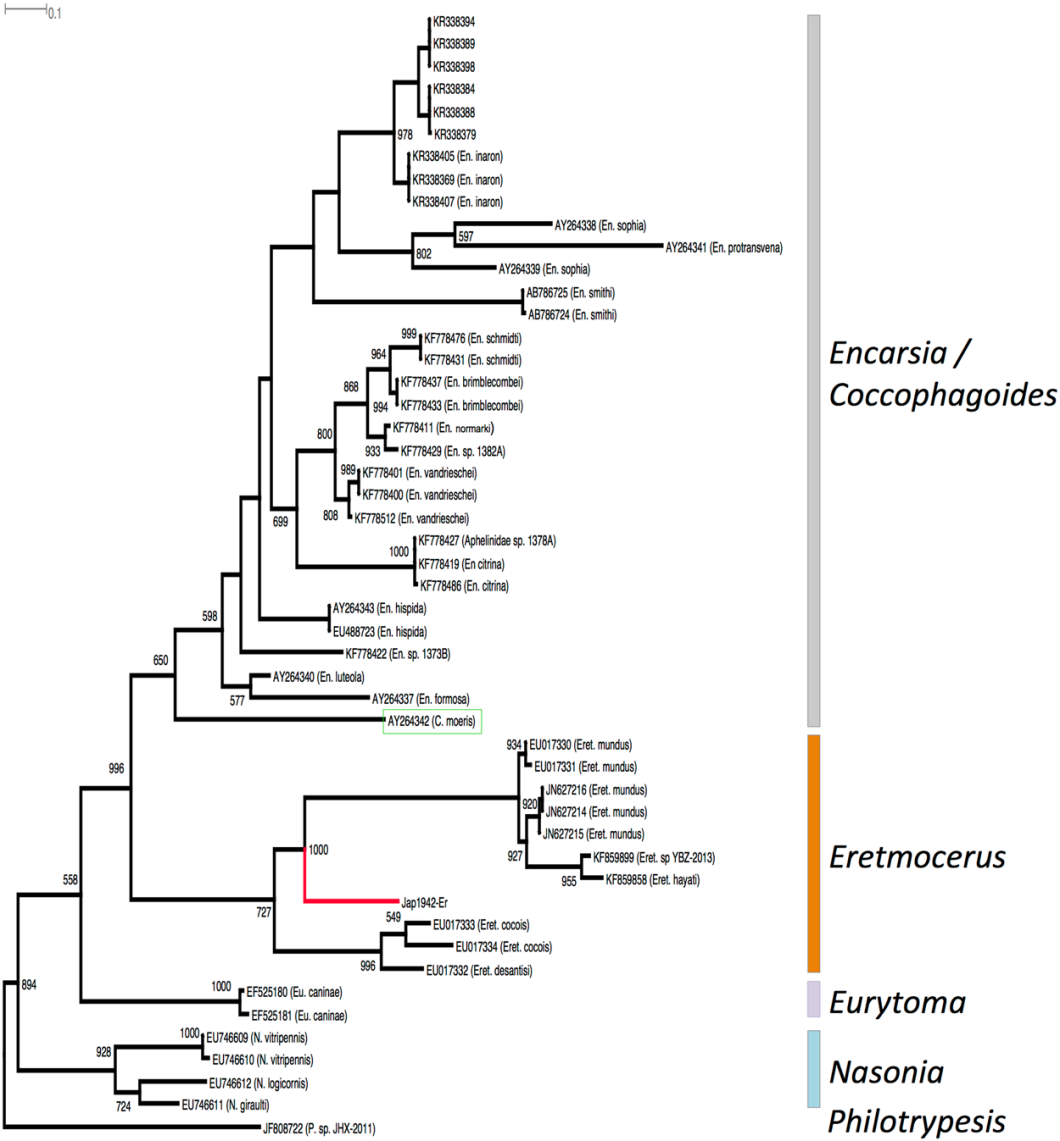
Phylogenetic placement of the unknown hymenopteran entity detected in the gDNA of the 1942 Japan *Bemisia* sp. The Hymenoptera is likely a parasitoid wasp species of the *Eretmocerus* genus based on partial (657 bp) mtCOI gene phylogeny, being placed within the ‘*Eretmocerus*’ clade (indicated by a red branch). The Aphelinidae wasps (‘*Encarsia*/*Coccophagoides*’ and ‘*Eretmocerus*’ species) formed sister clades with 99.6% node confidence. Green coloured box indicates *Coccophagoides moeris*. Outgroups used are *Eurytoma caninae* (Eurytomidae), *Nasonia* species (Pteromalidae) and *Philotrypesis* species (Pteromalidae). Node confidence of ≥50% (estimated from 1,000 bootstrap replications) are indicated.

Sequence identity of the 779 bp partial mtDNA COI C-terminal region matched the Aleyrodidae parasitoid *Eretmocerus cocois* (EU017333) at 90% identity, and between 88–89% sequence identity with 10 other publicly available (ie., through GenBank) but unpublished reports of *Eretmocerus* species including *Eret*. *desantisi*, *Eret*. *cocois*, *Eret*. *mundus*, *Eret*. *hayati*, and an unnamed *Eretmocerus* sp. YBZ-2013 (sequence identity to *Eret*. *hayati* = 98%) from China Xinjiang province (KF859899). Phylogenetic analysis (best substitution model: HKY85+G+I+F, proportion of invariable sites: estimated (0.387); number of substitution rate categories: 6; Gamma shape parameter: estimated (0.319); Lkl: −5517.437; AIC: 11260.873; K = 113) of the aligned 657 bp partial mtCOI sequences, and included various Chalcidoidea wasp species indicated that our unknown hymenopteran entity was from a separate evolutionary lineage basal to the sister clade that included *Eret*. *mundus*, *Eret*. *hayati*, and *Eret*. sp. YBZ-2013. The two sister clades of *Eret*. *mundus*-*Eret*. *hayati*-*Eret*. sp. YBZ-2013, and the Japanese 1942 hymenopteran individual were clustered with 100% bootstrap node support that indicated a shared most recent common ancestor. A third *Eretmocerus* basal sister clade included the Caribbean/New World species of *Eret*. *cocois* and *Eret*. *desantisi* from the French overseas territory of Guadeloupe^22^ (Fig. 3). All Aphelinidae wasps (i.e., *Eretmocerus* species including the unknown Japanese 1942 Hymenoptera, all *Encarsia*/*Coccophagoides* species) formed sister clades with each other and exhibited high (99.6%) bootstrap value.

## Discussion

To our knowledge, this is the first investigation into tritrophic interactions in historical *Bemisia* specimens. This was made possible by developing a protocol using low amounts of input double stranded gDNA (only 1.5 ng). This novel approach offers enormous potential to resolve existing taxonomic questions and also contribute to genome-wide surveys of individual historical *Bemisia* specimens, and can potentially be adopted for solving nomenclatural and taxonomic confusion in other cryptic species complexes. Since the synonymising of *Bemisia* species including *B*. *inconspicua* Quaintance (1900; collected in Florida, USA, synonymised by Russell^3^), *B*. *gossypiperda* Misra & Singh (1929; from India, synonymised by Russell^3^), and *B*. *emiliae* Corbett (1927; collected from Sri Lanka, synonymised by Mound and Halsey^2^ with ‘*B*. *tabaci*’; see also Martin and Mound^23^), genetic data based on partial mtDNA COI gene^4, 5, 8, 24^, and mating behaviour studies^4, 25–28^ have increasingly supported the recognition of *B*. *tabaci* as being a complex of cryptic species. By analysing historical specimens collected from the same geographic localities and at similar time frames prior to mass global commodity movements, it is possible to help ascertain historical species habitat boundaries, and ultimately contribute to rectify incorrectly synonymised species. In this respect, the cotype 1912 *B*. *emiliae* individual with partial mtCOI gene matching at high percentage (98–100%) with *B*. *tabaci* Asia II-7 may be seen as the next stage of solving *Bemisia* whitefly nomenclatural confusion, since the identification of the true *B*. *tabaci* ^6^. We further provided an example of cryptic species misidentification in the 1942 Japanese *Bemisia* species, and highlighted the on-going challenges in understanding the genetic diversity and species status of this global agricultural pest species complex.

Metagenomic compostions of various *B*. *tabaci* cryptic species have been investigated^29–31^ using gene-specific primers, with secondary endosymbionts such as *Arsenophonus*, *Cardinium*, and *Wolbachia* reported in Chinese *B*. *tabaci* ‘Asia II-7’ samples^29, 31^, while Bing *et al.* ^29^ also identified *Rickettsia* in their Asia II-7 material. In the 1912 *B*. *emiliae* individual, in addition to *Arsenophonus*, *Cardinium*, *Rickettsia* and *Wolbachia*, *Hamiltonella* was also detected. While we showed that both *B*. *emiliae* and the Japanese *Bemisia* species had, to a certain extent, similar P- and S-endobacterial compositions, there were also significant endosymbionts metagenomic profile differences. For example, the endosymbionts *Arsenophonus* and *Cardinium* were only detected in *B*. *emiliae*, as well as significant amount (14.9%) of Alphaproteobacteria, in contrast to only 4% detected in the 1942 *Bemisia* species (Table 2).

We identified two regions of a partial *wsp Wolbachia* gene (106 bp and 230 bp; GenBank KX714969) that showed between 98% and 99% sequence identity, to the *B*. *tabaci* Asia II-7 *Wolbachia wsp* gene (KJ600634) as reported by Ahmed *et al.* ^32^. The 230 bp partial *wsp* gene region from our historical 1942 specimen also showed high sequence homologies (99–100%) with diverse organisms, including a 99% sequence identity with the *Wolbachia wsp* gene from *B*. *afer*, and with native members of the *B*. *tabaci* species complex from China (e.g., Asia II3, Asia I, China I; Ji *et al.* ^33^), although reduced sequence identity (81.12–93.91%) were also detected between the historical *wsp* partial gene sequence and the partial sequences of ‘*B*. *tabaci*’ *Wolbachia* W4 and W6 strains (Table 3)^33^. Similarly, the 106 bp partial *wsp* gene that were identified also shared 100% sequence identity with the *Wolbachia* wsp gene of *B*. *afer* (AJ291370), and between 98.11–100% with *B*. *tabaci* (GU968901, JN315980, HQ404797) (Table 3), all of which were clustered at 73% on the same W1/W2 phylogenetic branch^33^. A lower sequence identity of 87.74% was also shared with the *Wolbachia wsp* gene (AJ291379) (Table 3) from a *B*. *tabaci* host that was shown to be phylogenetically basal to the W1-W6 *Wolbachia* strains reported by Ji *et al.* ^33^, and highlighted the difficulty of pin-pointing the identity of the *Wolbachia* strain(s) in our 1942 Miseq sequence data due to the short sequence nature of this partial gene region.

**Table 3:** Percentage (%) sequence identity of *Bemisia* species *Wolbachia* endosymbionts *wsp* partial gene (230 bp).

	Jap_1942	KJ648499	AJ291370	JN315980	FJ545748	KJ648498	HQ404797	GU968901	KJ648500	AJ291379	KJ648502	KJ648503	KJ648501
KJ648499	99.13												
AJ291370^†^	98.7 (100)	98.7											
JN315980^†^	98.7 (100)	98.7	100										
FJ545748	98.7	98.7	100	100									
KJ648498	98.7	98.7	100	100	100								
HQ404797^†^	98.26 (100)	98.26	99.57	99.57	99.57	99.57							
GU968901^†^	98.26 (98.11)	98.26	99.57	99.57	99.57	99.57	99.13						
KJ648500	95.65	96.52	96.96	96.96	96.96	96.96	96.52	96.52					
AJ291379^†^	94.78 (87.74)	95.65	96.09	96.09	96.09	96.09	95.65	95.65	97.39				
KJ648502	93.91	94.78	95.22	95.22	95.22	95.22	94.78	94.78	96.52	98.26			
KJ648503	93.91	94.78	95.22	95.22	95.22	95.22	94.78	94.78	96.52	98.26	97.39		
KJ648501	81.12	81.12	81.55	81.55	81.55	81.55	81.97	81.12	83.69	83.69	83.26	83.69	

The *Wolbachia wsp* partial gene from the historical 1942 *Bemisia* specimen (‘Jap_1942’) was most similar to KJ648499, which belonged to the W2 *Wolbachia* strain isolated from invasive (i.e., MED, MEAM1) and native (Asia I, Asia II3, China 2) *B*. *tabaci* cryptic species complex. ‘^†^’Indicates the five *Wolbachia* strains that had sufficient *wsp* gene sequence at the 5′ region to enable sequence identity comparison with the historical Japanese *Wolbachia wsp* gene (sequence identity acorss these 5′ end of 106 bp are indicated within parentheses).

**Note**: GenBank accession numbers provided for *Wolbacia* strains W1 (KJ648498), W2 (KJ648499), W3 (KJ648500), W4 (KJ648501), W5 (KJ648502), and W6 (KJ648503) are as reported by ref. 33.

Alternatively, it is also possible that the *Eretmocerus* parasitoid larva within the 1942 *Bemisia* nymph was itself infected with a different *Wolbachia* strain. Exploring the MiSeq data indentified a 189 bp *coxA* sequence (GenBank KX714965) that shared between 91% and 94.7% sequence identity with the majority of *B*. *tabaci* cryptic species *Wolbachia coxA* gene^32, 34^ but 100% sequence identity with *B*. *afer coxA* ST382 at this 189 bp region, as well as 100% sequence identity with the *coxA* partial gene of *Wolbachia* isolated from six diverse species including *Philaenus spumarius* (Hemiptera, Aphrophoridae) (KM377724), *Hypoponera* ant (Hymenoptera, Formicidae) (KF490396), *Macrosteles fascifrons* (Hemiptera, Cicadellidae) (HQ404763), *Sogatella furcifera* (Hemiptera, Delphacidae) (FJ713762), *Teleogryllus taiwanemma* (Orthoptera, Gryllidae) (DQ842303), and *Acraea encedon* (Lepidoptera, Nymphalidae) (DQ842269) (data not shown). Horizontal transfer of *Wolbachia* strains between phylogenetically distantly related arthropods has been reported previously^35–37^, and our partial *coxA* gene shared 100% sequence identity with the hemipteran (i.e., *P*. *spumarius*, *S*. *furcifera*) and orthopteran (i.e., *T*. *taiwanemma*) hosts that were also known to be present in Japan. The *coxA* locus between *Bemisia* species including *B*. *tabaci* cryptic species complex and *B*. *afer* species have been characterised (e.g., Ghosh *et al.* ^34^) and are highly similar. The 189 bp *coxA* partial gene from the 1942 *Bemisia* specimen captured only 23 SNPs of the total 40 SNPs present in the characterised *coxA* gene across a diverse groups of *B*. *tabaci* cryptic species, and shared 100% SNP identity with *B*. *afer* ST382 (Table 4). Given the partial *coxA* gene sequence matched both *B*. *afer*’s *Wolbachia* partial *coxA* ST382 sequence as well as non-*Bemisia* hosts, it suggests that the increase in *Wolbachia* sequences detected in the 1942 *Bemisia* specimen may well be due to infections in both the *Bemisia* nymph and the parasitoid larva.

**Table 4:** Comparison of single nucleotide polymorphism (SNP) profile of the *Wolbachia coxA* gene between known *Bemisia tabaci* cryptic species, *B*. *afer*, and the Japanese 1942 (JAP1942) *Bemisia* specimen.

Nucleotide position																				
	15	21	24	30	33	52	60	120	123	130	135	141	165	189	246	249	252	255	258	259
Consensus sequence	T	G	C	G	G	G	A	A	A	C	T	T	C	C	C	T	A	C	A	G
*coxA* 14	.	A	A	.	A	A	C	C	T	T	T	C	T	T	T	C	C	T	G	A
*B*. *afer* (ST382)	C	A	.	A	.	.	.	.	T	.	.	.	.	.	.	C	.	.	G	A
JAP1942 *coxA*	**?**	**?**	**?**	**?**	**?**	**?**	**?**	**?**	**?**	**?**	**?**	**?**	**?**	.	.	C	.	.	G	A
	**261**	**264**	**276**	**288**	**294**	**299**	**300**	**303**	**307**	**315**	**324**	**331**	**333**	**339**	**342**	**357**	**375**	**384**	**394**	**402**
consensus sequence	C	G	C	T	T	A	G	A	A	C	T	G	A	T	T	C	A	A	G	C
*coxA* 14	T	.	T	A	.	T	.	.	G	.	A	A	G	C	C	.	G	G	A	T
*B*. *afer* (ST382)	T	A	.	A	C	.	A	G	G	T	.	.	.	C	.	T	G	.	.	T
1942 *coxA*	T	A	.	A	C	.	A	G	G	T	.	.	.	C	.	T	**?**	**?**	**?**	**?**

*coxA*-14 sequences include *B*. *tabaci* SSA1-SG1, and *B*. *tabaci* SSA1-SG2 and are as reported in ref. 34. Consensus sequence identies are noted below. *coxA* missing regions for the 1942 Japanese *Bemisia* speciemen are between nucleotide positions 1 and 174, and from 364 to 402. Unknown SNP porfiles are indicated by ‘?’.

**Note**: consensus sequence SNP profiles were from identical coxA_88 seqeunce. *coxA* sequences aligned included: HQ404793, JQ013511, *B*. *tabaci* Q(ST116), *B*. *tabaci* Asia-I (ST378, ST385, ST395), *B*. *tabaci* Asia II-1 (ST389, ST390, ST391, ST392), *B*. *tabaci* Asia II-3 (ST396), *B*. *tabaci* Asia Ii-6 (ST393, ST394), *B*. *tabaci* Asia II-7 (ST378), *B*. *tabaci* Asia II-9 (ST384), *B*. *tabaci* China-1 (ST377, ST379, ST383), *B*. *tabaci* SSA1-SG5 (*coxA* 88), *B*. *tabaci* SSA1-SG3 (ST424, 425), *B*. *tabaci* Australia (ST380), and *B*. *afer* NG (ST427).

Primer efficacy issues may have contributed to the lack of detection of *Hamiltonella* by Bing *et al.* ^29^ and Zchori-Fein *et al.* ^31^, although host plant utilisation and environmental factors including adaptation to pesticides could also play a role in influencing endobacterial communities in *Bemisia* ^38–40^. *Hamiltonella* from *B*. *tabaci* MED/MEAM1 has been proposed to present no parasitoid-resistance due the inactivation of the APSE phage^41^. However, it can enhance hosts’ survival rates through an endosymbiont-mediated defense against parasitoid wasps in aphids^42^, In addition, *Hamiltonella* can also increase host growth rates in nutrient-poor environments^40^, or enhance reproductive rates and nymph growth rates, as reported for the invasive *B*. *tabaci* MED^39^. It is possible that host association with the *Hamiltonella* endosymbiont may therefore diminish in nutrient-rich environments (i.e., applications of crop fertilisers). Similary, applications of pesticides could result in the reduction of beneficial insects including parasitoids, and therefore reduce the benefits provided by this endosymbiont to its host (i.e., defense against parasitoid wasps). These anthropogenic factors often associated with the green revolution could have potential and indirect effects that contributed to the non-detection of *Hamiltonella* in present-day non-invasive *B*. *tabaci* Asia II-7 hosts as compared to the historical *B*. *emiliae* specimen. Given our current understanding, and the uncertainty about whether there are different strains of *Hamiltonella* being detected, it remains possible that this endosymbiont may be important for both nutrient benefit and parasitoid resistance, however, complete genome and the phylogenetic positions of *Hamiltonella* from *Bemisia* cryptic species including that from *B*. *emiliae* would be needed to better test the hypothesis postulated above. We would like to emphasise that our hypothesis has been postulated based on our very limited NGS data that were derived from very small historical samples. Globally, the near fixation of *Hamiltonella* has been reported in both the *B*. *tabaci* MEAM 1 and MED cryptic species^43^ within the ‘Africa/Middle East/Asia Minor’ clade^43^, however the level of association between *Hamiltonella* and other *B*. *tabaci* cryptic species lacks this level of knowledge. This hypothesis should be further tested through the analysis of additional museum *Bemisia* samples, especially via NGS methods as demonstrated from this study. While the endosymbiont *Fritschea bemisiae* (Chlamydiales) had been reported in the *B*. *tabaci* ‘New World’ species complex (previously Biotype A)^43^, this endosymbiont was not detected in both museum specimens analysed in this study. This again suggested potential differences in endobacterial metagenomic signatures between *B*. *tabaci* cryptic species from evolutionary diverse clades, as well as reflecting potential impact from both antropogenic (e.g. agricultural) activities and/or environmental/climatic differences.

Within the bacterial sequences from *B*. *emiliae*, a total of 291,123 sequences (30.1%) showed high homology to the proteobacteria *Acidovorax* genus, a genus that included highly-damaging agricultural species capable of damaging seeds and fruit crop (e.g., *A*. *citrulli* is capable of causing seeding blight and bacterial fruit blotch in cucurbits^44, 45^). The metagenomic analysis also identified a total of 40,391 sequences (4.2%) with homology to Verminephrobacter species (MR-RAST ID 4661244.3 and 4690946.3), which was closely related to *Acidovorax* bacteria. Verminephrobacter species has been reported only in the Lumbricidae earthworm *Eisenia foetida* so far. It is difficult to provide plausible explanations for the detection of significant number (i.e., greater number of sequences than the p-endosymbiont *Cand*. *P*. *aleyrodidarum*) of such sequences. Although environmental contamination remained a possibility, the 4^th^ instar *B*. *emiliae* larva was immersed in 100% ethanol for 24 hours prior to gDNA extraction, a process that would likely have reduced potential bacterial contaminations on the surface of the whitelfy nymph. This metagenomic bacterial signature is absent in the 1942 *Bemisia* specimen, and its detection in *B*. *emiliae* could be due environmental to factors or possibly of saprophytic origins.

The family Aphelinidae are predominantly parasitoid wasps of hemipteran sap-sucking insects, and includes the genera *Encarsia* and *Eretmocerus* that have *Bemisia* species among their hosts^22, 46–50^. Despite their importance as biological control agents of *Bemisia* whiteflies, species diversity in both *Encarsia* and *Eretmocerus* genera are likely to be underestimated. With >450 *Encarsia* species described (including 435 valid species at present) in this genus^51^, only 95 partial *Encarsia* mtCOI sequences (included 10 named *Encarsia* species: *En*. *luteola*, *En*. *formosa*, *En*. *hispida*, *En*. *citrina*, *En*. *vandrieschei*, *En*. *normarki*, *En*. *brimblecombei*, *En*. *schmidti*, *En*. *protransvena*, *En*. *inaron*, and five unnamed *Encarsia* species: *En*. sp. 1373B KF778422, Aphelinidae sp. 1378 A KF778427, *En*. sp. 1382 A KF778401, *En*. sp. 1369 A KF778420, *En*. sp. 1475 A KF778470) have been characterised at our aligned region (i.e., C-terminal region/3′-end) and are publicly available (GenBank nucleotide database, access 01-June-2016). Similary, only 18 mtDNA COI partial sequences representing seven (i.e., *Eret*. *hayati*, *Eret*. *mundus*, *Eret*. *emeritis*, *Eret*. *orchamoplati*, *Eret*. *cocois*, *Eret*. *desantisi*, *Eret*. sp. YBZ-2013) of 81 valid *Eretmocerus* species have been reported (GenBank nucleotide database, access 01-June-2016), and none originated from Japan, the likely origin of the *Eretmocerus* species detected in the 1942 *Bemisia* nymph host. Of these 18 reported *Eretmocerus* species mtCOI sequences, two belonged to *Eret*. *eremicus* (FM210161, FM210163) and six to *Eret*. *orchamoplati* (HQ660514, JF750711 to JF750715), and had the N-terminal region (i.e., 5′-end) of the mtCOI gene characterised at 460–651 bp. Sequence identities between the 1942 Japanese *Eretmocerus* species and *Eret*. *eremicus* or *Eret*. *orchamoplati* were both at 85% for this 5′-end partial mtCOI gene region, further ruling out them as candidates.

**Figure 4:**
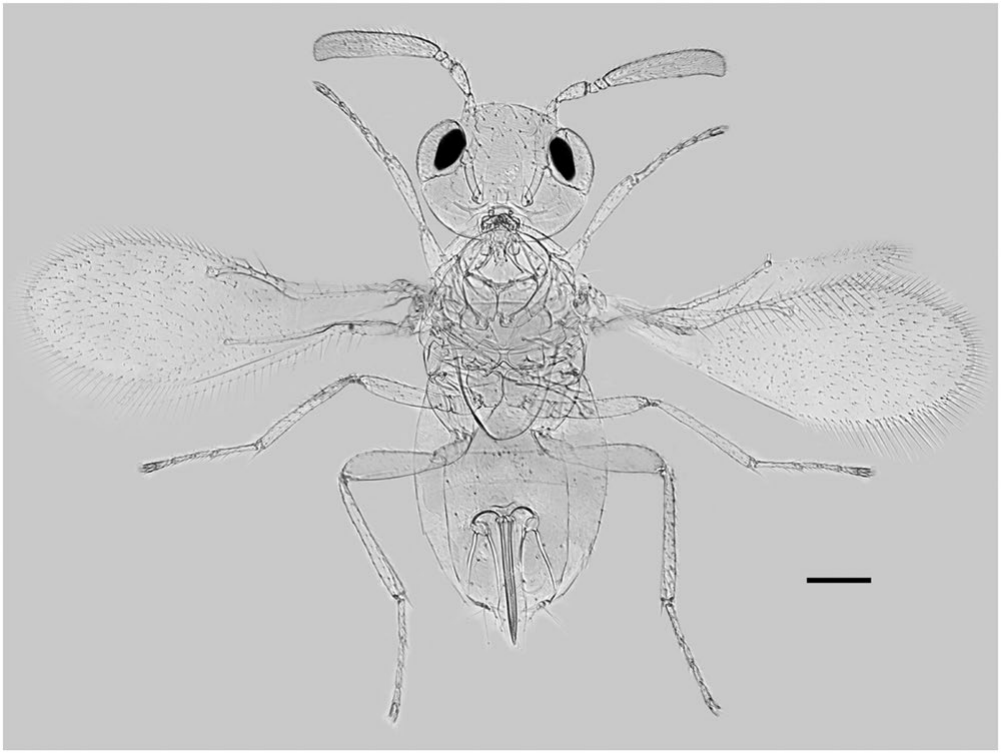
*Eretmocerus furuhashii* Rose: composite image from 4 female paratype specimens (Irvine 108, Orange Co. California, USA. 29.vii.1982 ex *Parabemisia myricae*. Ferrentino + Woolley col. BMNH(E) 1996-136). Scale bar = 0.1 mm. The image was produced using AutoMontage (Synoptics Ltd, Cambridge, UK) stacking imaging software on a set of images taken with a Q-Imaging RoHS camera and processed using Adobe Photoshop CC version.

Three species of *Eretmocerus* are currently known from Japan^51^: *Eret*. *aleurolobi* (Ishii 1938), *Eret*. *furuhashii* (Rose and Zolnerowich 1994) and *Eret*. *serius* (Silvestri 1927), but none has yet been sequenced for mtCOI genes. *Eret*. *aleurolobi* has only been recorded from *Aleurolobus marlatti*. *Eret*. *serius* is a well-known parasitoid of *Aleurocanthus* species in the Oriental region, with two published records from *B*. *tabaci*, neither of which appears to be reliable^52, 53^. The third species, *Eret*. *furuhashii* is known only from *Parabemisia myricae*. However, Hoelmer and Goolsby^54^ record ‘*Eret*. near *furuhashii*’ as one of the species introduced from Taiwan into USA against *B*. *tabaci*. This identification would have been made by Rose, who clearly considered this *B*. *tabaci* parasitoid close to, but distinct from, *Eret*. *furuhashii* which he had co-described in 1994. There is thus strong circumstantial evidence that our historical *Eret*. sp. from Japan is this undescribed species from Taiwan that Rose designated ‘near *furuhashii*’. In China, at least 19 aphelinid wasps have been recorded to parasitise *Bemisia*, with *Eret*. sp. nr. *furuhashii* being the most abundant, followed by *En*. *bimaculata* (Heraty and Polaszek), being responsible for 15–87.3% parasitisim in agricultural crops^55–57^. Given that the partial mtCOI phylogeny did not support our unknown parasitoid as being an *Encarsia* species, it would rule out *En*. *bimaculata*, leaving *Eret*. sp. nr. *furuhashii* (Fig. 4) as the most probable candidate. Two further pieces of supporting evidence that *Eret*. sp. near *furuhashii* is the likely candidate came from the partial mtCOII (cytochrome oxidae subunit II) gene (424 bp; GenBank KX714953) and the 28 s rRNA gene (GenBank KX714966) assembled and identified from the 1942 Japanese *Bemisia* nymph specimen, where these two partial hymenopteran genes (i.e., mtCOII, 28 s rRNA) matched *Eret*. sp. nr. *furuhashii* from Sanya China, between the mtCOII at nucleotide positions 64 and 332 (i.e., JF820015, 266 bp) with 98.14% sequence identity, and between the 28 s rRNA at nucleotide positions 4,348 and 4,804 (i.e., JF899345, 457 bp) with 100% sequence identity.

Although intriguing, it remains to be seen whether the 1942 Japanese native *Bemisia* species is a natural host of *Eret*. sp. nr. *furuhashii*, and whether the abundance of this aphelinid wasp is the same in Japan as in China. The identity of the *Eretmocerus* species that parasitized the Japanese 1942 *Bemisia* nymph will for now remain unconfirmed and intra-species genetic diversity survey, followed by morphological and molecular characterisation of aphelinid wasps in this native Japanese *Bemisia* species will be needed for its future identification.

Our study demonstrated the possibility of investigating tritrophic interactions between host, parasitoid and endosymbionts in museum specimens from just 1.5 ng of double stranded gDNA. While Tin *et al.* ^58^ demonstrated between 14–220 ng of gDNA would be sufficient for genome-wide SNP analysis via the Restriction-Associated DNA sequencing (i.e., RAD-Tag/RADseq) method for museum specimens (collected from 1910 to 1976), our protocol further reduced the required gDNA for NGS methods to approximately one-tenth of the 14 ng used by Tin *et al.* ^58^. Our method also overcame the challenge of working with highly fragmented gDNA starting material without performing an initial ‘end-repair’ step^59^ that may lead to the loss of gDNA. Although non-destructive extraction of gDNA is possible from a range of small insects^60^ including Aphelinidae wasps^47^, it was not attempted for these *Bemisia* museum specimens, and could be adopted to provide a non-destructive NGS protocol for the recovery of these historical specimens. Our method described here will offer significantly improved opportunity to better investigate the genomics of historical specimens, and together with new genome-wide SNP generating methodologies that have been developed^58^, will deepen our knowledge regarding how current ecological and climatic conditions as well as anthropogenic factors have impacted on species historical distributional range and their bacterial metacommunity compositions.

## Material and Methods

A single *Bemisia emiliae* pupa (cotype) was collected from Hakgala, Sri Lanka (formerly Ceylon), by E. E. Green in May 1912. It was formerly described 14 years later^61^. The specimen was deposited at the Smithsonian Institution, Washington D.C. The second sample is a single whitefly 4^th^ instar nymph collected by S. Kanda in Muroto, Shikoku, Japan on 08-Aug-1942 and morphologically identified as *B*. *tabaci*. All samples were made available for use in this study by Greg Evans, USDA APHIS NIS.

The extraction protocol consisted of first placing the pupa in 1,000 µL of analytical grade 99.9% ethanol for 24 hours in a sterile 1.5 mL Eppendorf tube. The ethanol was then removed and the specimen and air-dried for 15 minutes at room temperature. Once dried, the pupa was crushed in a sealed P200 sterile pipette tip and extracted using a modified protocol that combined the Qiagen Blood and tissue DNA extraction kit and the Zymo Research DNA concentrator protocol. The protocol was modified to improve the quality and yield of DNA extracted. It involved digesting the crushed pupa in 196 µL of AL (Qiagen) and 4 µL of proteinase K (Qiagen; >600 mAU/mL) for 24 hours at 56 °C. At the end of the digestion step, 4 µL of RNase A (Qiagen; 100 mg/mL) was added and incubated at room temperature for 5 minutes. The digestion buffer with the pupa was pulse-spun and 200 µL of wash buffer AW1 (Qiagen) was added. The liquid was then transferred to the Zymo Research genomic DNA concentrator column, and centrifuged as per the Zymo Research genomic DNA concentrator protocol (10,000 g, 1 min). The washing step was repeated and the gDNA was finally eluted in 15 µL of Buffer EB (Qiagen, Cat. # 19086). We used 2 µL of the eluted gDNA to estimate DNA concentration using Qubit v2.0 (Life Technologies, Grand Island, NY) dsDNA HS assay. The remaining amount of eluted gDNA (*ca*. 3.98 ng (*B*. *emiliae*); *ca*. 24.01 ng (1942 ‘*Bemisia*’)) in Elution Buffer (~12.5 µL) was allowed to evaporate at room temperature for 16 hours (in a sterile fume hood, uncapped, but loosely covered by a piece of clean Kimwipe tissue). The dried gDNA from the pupa was then re-suspended in 5 µL nuclease-free water (Qiagen, Cat.129114) for 60 minutes at room temperature.

### Preparation and normalisation of Nextera XT library to 2 nM of amplicon molecules

Although the Nextera XT protocol recommended only 1.0 ng of double stranded gDNA as starting input material, we used 1.5 ng of double stranded gDNA for very small museum specimens such as *Bemisia* nymphs, to compensate for the likely presence of very fragmented gDNA (i.e., <50 bp) and to allow for targeting of slightly larger fragments for 2 × 300 bp PE sequencing. The amount of 1.5 ng of double stranded gDNA was sampled from each of *B*. *emiliae* and the 1942 *Bemisia* specimens and processed using the Nextera XT DNA Library Preparation kit (Illumina, Cat. # FC-131-1096). Individual *Bemisia* pupae gDNA was tagmented (tagged and fragmented) by the Nextera XT transposome. The tagmented gDNA was amplified in a limited cycle PCR reaction to add indexes and Illumina adapter sequences (Illumina, Cat. # FC-131-2003) for sample tracking and cluster formation. For each *Bemisia* specimen, the amplified library was purified and size-selected using AMPureXP beads (Beckman Coulter, Cat. # A63881). Purified libraries were then quantified by Qubit dsDNA HS assay and the size distribution checked by HS D1000 screentape assay (Agilent, Cat. # 5067–5584) on an Agilent 2200 Tapestation. The insert sizes of our gDNA libraries were determined to range between 160–500 bp for *B*. *emiliae* and between 154–1,234 bp for the 1942 ‘JpL’ *Bemisia* species, and both have a peak fragment size of 256 bp. The Illumina Nextera XT libraries were then normalized to a final concentration of 2 nM, denatured and diluted to a final concentration of 10 pM and combined with a Phi X control library (Illumina, Cat. # FC-110-3001), spiked in at 2.5%. The Illumina libraries were then sequenced on an Illumina MiSeq sequencer using a MiSeq reagent kit v3, 600 cycles (Illumina, Cat. # MS-102-3003) to perform a 2 × 301 bp paired-end sequencing run.

We used Geneious version 8.0.5 (Biomatters Ltd, Auckland, NZ) to assemble of the full mitogenome of *B*. *emiliae*, using the *Bemisia tabaci* cryptic species Asia I mitogenome (KJ778614)^11^ as a reference. We used Illumina reads with quality checking performed within Geneious version 8.0.5 for mitogenome assembly and annotations. The assembly parameters consisted of 10% maximum mismatches per read, minimum overlap of 25 bp, maximum gap size of 3 bp, and minimum overlap identify of 80%. The mitogenome annotation was conducted using MITOS^62^, with manual fine-tuning of putative start codon position for all protein coding genes within Geneious. Pairwise mitogenome alignments between the 1942 ‘*B*. *tabaci*’, *B*. *emiliae*, and *B*. *tabaci* Asia I (KJ778614) were performed using MAFFT v7.017^63^ within the Geneious version 8.0.5 program and implementing default parameters (Algorithm: Auto; Scoring matrix: 200 PAM/k = 2; Gap open penalty: 1.53; Offset value: 0.123). To quantify microbial community compostions in the gDNA of both historical *Bemisia* specimens, paired-end sequence reads were analysed using MG-RAST metagenomic analysis server version 3.6. Analyses of gDNA from both 1912 *B*. *emiliae* and 1942 ‘*B*. *tabaci*’ samples were carried out using the default settings. Output sequences best matching hymenopteran mtDNA genes were used as template for re-assembly of contigs in Geneious version 8.0.5. MG-RAST metagenomic analysis results can be accessed from the MG-RAST website http://metagenomics.anl.gov using the accession numbers 4681440.3 and 4690946.3 for the 1912 *B*. *emiliae* and the 1942 *Bemisia* species, respectively.

We conducted a phylogenetic analysis to infer species relationships of the unknown Hymenoptera to selected Chalcidoidea parasitoid wasp species belonging to six genera (i.e., *Eretmocerus*, *Nasonia*, *Philotrypesis*, *Coccophagoides*, *Encarsia*, *Eurytoma*; Supplementary Table 2), based on partial mtCOI gene as obtained from GenBank nucleotide database (accessed 01-Jun-2016). Sequences were imported into CLC Sequence Viewer version 7.6 (Qiagen Aarhus A/S) and followed by alignment using default settings (Gap open cost = 10; Gap extension = 1.0). Alignment of the partial mtCOI gene is generally straight forward across diverse insect groups as it typically does not involve INDELs. Trimming of sequences therefore only involved removal of flanking 5′ and 3′ regions that extended beyond the region and length of interest. Trimmed sequences (657 bp) were used to generate a Maximum Likelihood phylogenetic tree using PhyML 3.0 http://atgc-montpellier.fr/phyml/, using the ‘automatic model selection’ option, followed by 1,000 bootstrap replications to estimate node confidence. The phylogenetic tree was then visualized in Dendroscope (Hudson and Scornavacca 2012) version 3.2.10 www.dendroscope.org.

### Identification of *Wolbachia coxA* and *wsp* partial gene sequences

The two regions of the *Wolbachia wsp* partial gene (106 bp, 230 bp: GenBank KX714969) were compared to sequences available in GenBank using Blastn search, and aligned and trimmed in Geneious version 8.0.5 prior to estimating the leve of sequence identity (Table 3). The *coxA* database was accessed (9-July-2016) and aligned with the 189 bp partial *coxA* gene sequence (GenBank KX714965) identified from the 1942 *Bemisia* specimen using Geneious version 8.0.5 to identify SNP patterns (Table 4).

### Identification of the parasitoid mtDNA and ribosomal (18S–28S) gene sequences

We assembled the complete 18 s rRNA/ITS1/5.8 s rRNA/ITS2/28 s rRNA genes of the parasitoid larva from the 1942 *Bemisia* 4^th^ instar nymph by using the *Eret*. sp. nr. *furuhashii* partial 28 s rRNA sequence (JF820005) from GenBank as reference sequence. The contig obtained was characterised for ribosomal RNA subunits using the web-based RNAmmer 1.2 Server^64^ to predict the presence of 5 s/8 s, 16 s/18 s and 23 s/28 s ribosomal RNA. Contig assembly for various mtDNA genes were based on partial hymenopteran mtDNA gene regions identified by MG-RAST. These partial gene regions were subsequently used as reference sequences for contig assembly in Geneious version 8.0.5, using default settings as described for the assembly of the 1912 *B*. *emiliae* and the 1942 *Bemisia* mitogenomes. To ensure that as many of as possible of known Chalcidoidea mtDNA genes were identified in our 1942 *Bemisia* specimen MiSeq Nextera XT library, we also used the *Nasonia longicornis* partial mtDNA genome (EU746612) previously reported by Oliveira *et al.* ^65^ as a reference genome in mining for mitochondrial DNA genes.

### Ascertaining historical *Bemisia* gDNA quality against published *B*. *tabaci* genome

The paired-end reads corresponding respectively to *B*. *emiliae* and *B*. sp. ‘JpL’ were mapped to the *B*. *tabaci* (MEAM1) genome http://www.whiteflygenomics.org/cgi-bin/bta/blast.cgi (last accessed 14-November-2016) using the Burrows-Wheeler Aligner BWA v.0.7.12^66^, with the defaults parameters. The mapped reads were converted from sam to bam format and checked for mapping quality using SAMtools^67^. The percentage of read mapping in the case of *B*. *emiliae* is 80.04%, from which 77.44% of reads were properly paired, whereas it was respectively 53.92% and 49.95% for *B*. sp. ‘JpL’.

## Electronic supplementary material

Supplementary Information
