# Supplementary material for: Novel molecular approach to define pest species status and tritrophic interactions from historical Bemisia specimens
Source: Sci Rep. 2017 Mar 27;7:429. doi: 10.1038/s41598-017-00528-7 (PMC5428565; doi:10.1038/s41598-017-00528-7)
Supplement: Supplementary file 1 — Supplementary Information [file 41598_2017_528_MOESM1_ESM.pdf]

Novel molecular approach to define pest species status and tritrophic interactions from historical *Bemisia* specimens

Tay WT[1], Elfekih S[1], Polaszek A[2], Court LN[1], Evans, GA[3], Gordon KHJ[1], De Barro PJ[4]

1. CSIRO, Black Mountain Laboratories, Clunies Ross Street, ACT 2601, Australia
2. Natural History Museum, London United Kingdom
3. USDA APHIS NIS, BARC-West, Beltsville, Maryland, United States of America,
4. CSIRO, Brisbane 4001, Queensland, Australia

Correspondence:

W. T. Tay E-mail: weetek.tay@csiro.au **Tel:** +61-(0)2-6246 4286

Supplementary Fig. 1: Diagrammatic representation of pair-end contig assembly spanning the tRNA^{Met} (nucleotide positions 59 to 126), tRNA^{Lys} (nucleotide positions 186 to 233), and the mtCOI (from nucleotide position 362) genes of the detected *Eretmocerus* parasitoid wasp within the 1942 'JpL' *Bemisia* species. The 48bp tRNA^{Lys} is located within five NGS fragments (fragments 2-6), while fragments 1 and 2 formed pair-end contigs that also included the parasitoid wasp's mtCOI gene.

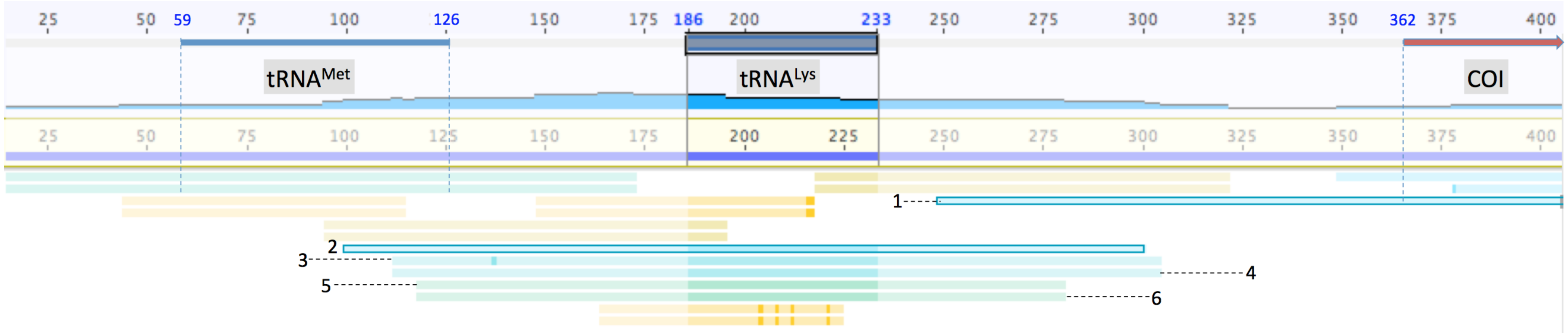

Supplementary Table 1: Mitochondrial DNA gene contents including orientation for *B. emiliae* and the 1942 Japanese *Bemisia* species. Missing genes in the 1942 *Bemisia* sample are indicated by '?'. Orientation of genes are indicated by ← or → arrows. The anticodon of the tRNA^{Met} of the Japanese *Bemisia* species is (TAT).

	Bemisia emiliae				**tRNA in**	*Bemisia* sp. (Japan 1942)			
**Name**	**Start**	**End**	**Length (bp)**	**Direction**	**Suppl. Fig.1**	**Start**	**End**	**Length (bp)**	**Direction**
COI	1	1,542	1,542	forward		1	1,542	1,542	→
trnL2(taa)	1,538	1,602	65	forward	1	1,538	1,602	65	→
COII	1,603	2,286	684	forward		1,603	2,286	684	→
trnK(ttt)	2,267	2,333	67	forward	2	2,267	2,333	67	→
ATP8	2,350	2,595	246	forward		2358	?	>120	→
ATP6	2,585	3,235	651	forward		?	?	?	?
trnS1(tct)	3,263	3,324	62	reverse	3	?	?	?	?
trnE(ttc)	3,333	3,396	64	reverse	4	?	?	?	?
ND5	3,405	5,132	1,728	reverse		<3,996	5,140	>1,145	←
trnF(gaa)	3,415	3,481	67	reverse	5	?	?	?	?
trnH(gtg)	5,133	5,200	68	reverse	6	5,141	5,206	66	←
ND4	5,201	6,493	1,293	reverse		5,207	6,493	1,287	←
NAD4L	6,490	6,774	285	reverse		6,490	6,774	285	←
trnT(tgt)	6,776	6,839	64	forward	7	6,775	6,838	64	←
trnP(tgg)	6,840	6,901	62	reverse	8	6,839	6,900	62	←
NAD6	6,936	7,382	447	forward		6,902	7,381	480	→
CYTB	7,383	8,516	1,134	forward		7,382	8,518	1,137	→
trnS2(tga)	8,515	8,570	56	forward	9	8,518	8,572	55	→
NAD1	8,588	9,490	903	reverse		8,591	9,493	903	←
trnL1(tag)	9,506	9,575	70	reverse	10	9,506	9,574	69	←
rrnL	9,603	10,784	1,182	reverse		9,599	>1,0215	>617	←
trnV(tac)	10,785	10,851	67	reverse	11	10,785	10,852	68	←
trnD(gtc)	10,856	10,931	76	reverse	12	10,865	10,941	77	←
trnQ(ttg)	10,937	11,000	64	reverse	13	10,947	11,011	65	←
rrnS	11,005	11,756	752	reverse		11,018	>11,726	>709	←
trnN(gtt)	11,918	11,984	67	reverse	14	?	?	?	?
trnR(tcg)	11,986	12,055	70	reverse	15	?	?	?	?
trnA(tgc)	12,059	12,127	69	reverse	16	?	?	?	?
NAD3	12,131	12,484	354	reverse		?	?	?	?
trnG(tcc)	12,486	12,548	63	reverse	17	?	?	?	?
COIII	12,595	13,374	780	reverse		<13,102	13,377	>276	←
trnI(gat)	14,218	14,279	62	forward	18	?	?	?	?
trnM(cat)	14,281	14,349	69	forward	19	13,600	13,667	68	→
NAD2	14,359	15,318	960	forward		14,057	15,016	960	→
trnW(tca)	15,317	15,383	67	forward	20	15,015	15,081	67	→
trnY(gta)	15,382	15,444	63	reverse	21	15,080	15,142	63	←

trnC(gca)	15,447	15,513	67	reverse	22	15,145	15,211	67	←

Supplementary Table 2: GenBank accession numbers of selected representative Chalcidoidea wasps partial mitochondrial DNA cytochrome oxidase subunit I (mtCOI) gene used to infer phylogenetic position of the unknown Hymenoptera mtCOI gene detected in the 4th instar nymph of a 1942 Japanese *Bemisia* species. Strain (s), isolate (i), haplotype (h) and voucher specimen (vs) are indicated.

	GenBank	Genus	sp.	strain/isolate/ haplotype/voucher specimen	s/i/ h/vs	Family
1	EU017332.1	*Eretmocerus*	*desantisi*	GP2411_3	i	Aphelinidae
2	EU017333.1	*Eretmocerus*	*cocois*	GP2411_4	i	Aphelinidae
3	EU017334.1	*Eretmocerus*	*cocois*	44P	i	Aphelinidae
4	JN627216.1	*Eretmocerus*	*mundus*	E3	i	Aphelinidae
5	JN627214.1	*Eretmocerus*	*mundus*	E1	i	Aphelinidae
6	JN627215.1	*Eretmocerus*	*mundus*	E2	i	Aphelinidae
7	KF859899.1	*Eretmocerus*	sp. YBZ-2013	YBZ-2013	s	Aphelinidae
8	EU017330.1	*Eretmocerus*	*mundus*	GDEL_14	s	Aphelinidae
9	EU017331.1	*Eretmocerus*	*mundus*	GDEL_22	s	Aphelinidae
10	KF859858.1	*Eretmocerus*	*hayati*			Aphelinidae
11	EU746611.1	*Nasonia*	*giraulti*	RV2	s	Pteromalidae
12	EU746612.1	*Nasonia*	*longicornis*	IV7	s	Pteromalidae
13	EU746609.1	*Nasonia*	*vitripennis*	AsymC	s	Pteromalidae
14	EU746610.1	*Nasonia*	*vitripennis*	HiCD12	s	Pteromalidae
15	JF808722.1	*Philotrypesis*	sp. JHX-2011	pphisI1_hn		Agaonidae
16	AY264342.1	*Coccophagoides*	*moeris*			Aphelinidae
17	AY264340.1	*Encarsia*	*luteola*			Aphelinidae
18	AY264337.1	*Encarsia*	*formosa*			Aphelinidae
19	KF778422.1	*Encarsia*	sp. 1373B	1373B	i	Aphelinidae
20	EU488723.1	*Encarsia*	*hispida*			Aphelinidae
21	AY264343.1	*Encarsia*	*hispida*			Aphelinidae
22	KF778419.1	*Encarsia*	*citrina*	1367A	i	Aphelinidae
23	KF778427.1	*Aphelinidae*	sp.	1378A	i	Aphelinidae
24	KF778486.1	*Encarsia*	*citrina*	1720A	i	Aphelinidae
25	KF778512.1	*Encarsia*	*vandrieschei*	2580A	i	Aphelinidae
26	KF778400.1	*Encarsia*	*vandrieschei*	1309A	i	Aphelinidae
27	KF778401.1	*Encarsia*	*vandrieschei*	1312A	i	Aphelinidae
28	KF778429.1	*Encarsia*	sp. 1382A	1382A	i	Aphelinidae
29	KF778411.1	*Encarsia*	*normarki*	1357A	i	Aphelinidae
30	KF778433.1	*Encarsia*	*brimblecombei*	1389A	i	Aphelinidae
31	KF778437.1	*Encarsia*	*brimblecombei*	1400A	i	Aphelinidae
32	KF778431.1	*Encarsia*	*schmidti*	1387A	i	Aphelinidae
33	KF778476.1	*Encarsia*	*schmidti*	1500A	i	Aphelinidae
34	AB786724.1	*Encarsia*	*smithi*	T-J1	h	Aphelinidae
35	AB786725.1	*Encarsia*	*smithi*	T-J2	h	Aphelinidae

36	AY264339.1	*Encarsia*	*sophia*	M93003	s	Aphelinidae
37	AY264341.1	*Encarsia*	*protransvena*			Aphelinidae
38	AY264338.1	*Encarsia*	*sophia*	M95107	s	Aphelinidae
39	KR338384.1	*Encarsia*	*inaron*	INIS6	i	Aphelinidae
40	KR338379.1	*Encarsia*	*inaron*	INIS1	i	Aphelinidae
41	KR338388.1	*Encarsia*	*inaron*	INIS10	i	Aphelinidae
42	KR338398.1	*Encarsia*	*inaron*	INIT10	i	Aphelinidae
43	KR338389.1	*Encarsia*	*inaron*	INIT1	i	Aphelinidae
44	KR338394.1	*Encarsia*	*inaron*	INIT6	i	Aphelinidae
45	KR338407.1	*Encarsia*	*inaron*	INIR5	i	Aphelinidae
46	KR338405.1	*Encarsia*	*inaron*	INIR3	i	Aphelinidae
47	KR338369.1	*Encarsia*	*inaron*	INAZ1	i	Aphelinidae
48	EF525180.1	*Eurytoma*	*caninae*	H168	vs	Eurytomidae
49	EF525181.1	*Eurytoma*	*caninae*	H169	vs	Eurytomidae
